# Properties and alcohol sensing applications of quasi-2D (PEA)_2_(MA)_3_Sb_2_Br_9_ thin films

**DOI:** 10.1186/s11671-023-03806-8

**Published:** 2023-02-20

**Authors:** Chien-Min Hun, Lung-Chien Chen

**Affiliations:** Department of Electro-Optical Engineering, National Taipei University of Technology, Taipei, 10608 Taiwan

**Keywords:** Lead-free perovskite, (PEA)_2_MA_3_Sb_2_Br_9_, Quasi-2D material, Alcohol detectors

## Abstract

We fabricated an alcohol detector based on (PEA)_2_(CH_3_NH_3_)_3_Sb_2_Br_9_ ((PEA)_2_MA_3_Sb_2_Br_9_) lead-free perovskite-like films. The XRD pattern revealed that the (PEA)_2_MA_3_Sb_2_Br_9_ lead-free perovskite-like films exhibited a quasi-2D structure. The optimal current response ratios are 74 and 84 for 5 and 15% alcohol solutions, respectively. When the amount of PEABr decreases in the films, the conductivity of the sample in ambient alcohol with a high alcohol concentration solution increases. The alcohol was dissolved into water and carbon dioxide due to the catalyst effect of the quasi-2D (PEA)_2_MA_3_Sb_2_Br_9_ thin film. The rise and fall times for the alcohol detector were 1.85 and 0.7 s, respectively, indicating that the detector was suitable.

## Introduction

Organic–inorganic lead halide MAPbX_3_ (MA = CH_3_NH_3_, X = Cl, Br, and I) perovskite is widely used in solar cells, light-emitting diodes (LEDs), detectors, and lasers. Due to its excellent optoelectronic properties in exciton binding energy, carrier mobility, carrier diffusion lengths, light absorption range, and tunable optical band gap by using composition variation, perovskite is suitable for the solar spectrum. Therefore, perovskite solar cells have quickly attracted widespread attention in the field of high-efficiency photovoltaic cells [1–10]. For the past decade, the power conversion efficiency of perovskite solar cells has rapidly increased from less than 5% to 25.7% [11–14]. Although the efficiency has been significantly improved, due to the considerable influence of the environment on the efficiency of the device, such as the humidity in the ambient air, the stability of the device cannot be significantly improved for a long time [15–17].

The perovskite materials also applied in gas sensing field owing to their fascinating catalytic property caused by a lot of defects and oxygen vacancies [18, 19]. In addition, the toxicity of lead-based perovskites may also be a major obstacle for sensor applications. The lead toxicity problem of perovskite gas sensors is more important than that of solar cells and LEDs because the gas sensor film needs to be exposed to air for a long time and because of the environmental impact on the sensor performance. Recently, a lead (Pb)-free material with the similar structure as perovskite has emerged: A_3_B_2_X_9_ (A = MA or Cs; B = Bi or Sb; X = I, Br, and Cl). The bismuth and antimony halide materials are typical perovskite-like material with no toxicity, such that they are potential materials for optoelectronic and biochemical applications [20–24]. Since Bi is a low-toxicity element adjacent to Pb, A_3_Bi_2_X_9_, which has better stability than MAPbI_3_ perovskite [24], can be used as a substitute for Pb-based perovskite materials. Thus, it is a potential new light absorbing material. As an element belonging to the same main group as Bi, Sb has a similar arrangement and distribution of outer electrons to Bi and has been used as a substitute material for Pb [25].

However, to our knowledge, these low-toxicity perovskite materials have not been investigated in the field of gas sensors. Considering that this type of material can solve the aforementioned problems, after exploring related materials, we demonstrate for the first time a new application of lead-free perovskite (PEA)_2_MA_3_Sb_2_Br_9_ in alcohol (C_2_H_5_OH) detection. Compared with the use of MA^+^, the introduction of PEA^+^ enhances the hydrophobicity and stability of perovskite materials [26–28]. In this study, the performance and results show that the (PEA)_2_MA_3_Sb_2_Br_9_ alcohol detector exhibits relatively excellent performance.

## Experiments

### Sensor Manufacturing Process

**Fig. 1:**
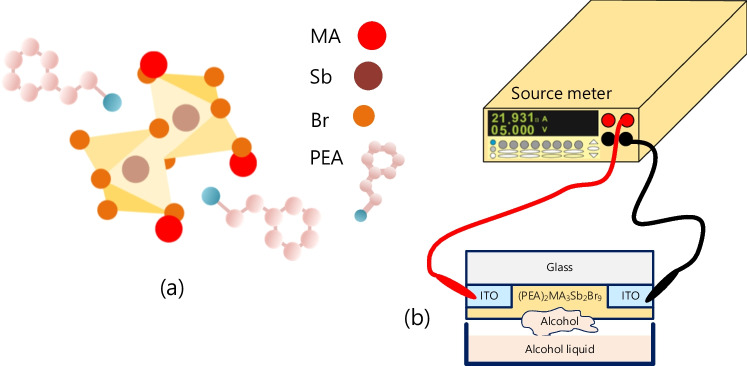
**a** Structure of (PEA)_2_MA_3_Sb_2_Br_9_ and **b** sketch of the experimental set up

The precursors SbBr_3_, MABr, and PEABr were dissolved in 1 mL of DMF and stirred at RT for 30 min at 750 rpm to obtain (PEA)_2_MA_3_Sb_2_Br_9_ perovskite solutions. The structure of (PEA)_2_MA_3_Sb_2_Br_9_ is shown in Fig. 1a. The recipe is listed in Table 1. A total of 50 µL of the prepared perovskite solution was dropped on glass with an ITO pattern, and a two-stage spin coating was performed using a spin coater. The first stage was 1000 rpm for 10 s, and the second stage was 5000 rpm for 20 s to form perovskite films. Additionally, 100 µL of toluene was dropped on the surface and then baked on a hot plate at 100 °C for 10 min.

**Table 1:** Recipe of various (PEA)_2_MA_3_Sb_2_Br_9_ films

Sample	SbBr_3_ (mmol/ml)	MABr (mmol/ml)	PEABr (mmol/ml)
PEA1	1	0.8	0.4
PEA2	1	0.9	0.2
PEA3	1	0.975	0.05
MA_3_Sb_2_Br_9_	1	1	0

### Measurement

The (PEA)_2_MA_3_Sb_2_Br_9_ perovskite sensor was placed in air, and a voltage from 0 to 10 V was applied to record the background current. After that, the (PEA)_2_MA_3_Sb_2_Br_9_ sensor was placed in an alcohol environment, and after waiting for 30 s, a voltage of 0 to 10 V was applied again, and its current–voltage (*I*–*V*) characteristics were recorded. Figure 1b sketches the experimental set up. In alcohol response measurement, the definition of the normalized responsivity ratio (R) of the sensor is as follows:

1 $$R=\frac{\Delta I}{{I}_{0}}=\frac{\left(I-{I}_{0}\right)}{{I}_{0}}$$

where *I*_o_ is the background current and I is the signal current under ambient alcohol at 10 V. To observe the different sensitivities of the sensor to alcohol during a period of time, we measured the current–time (*I*–*T*) characteristics of the (PEA)_2_MA_3_Sb_2_Br_9_ alcohol sensors. The applied voltage is 10 V. The first step is to put the sensor in the air, and after 30 s, we put the sensor in an alcohol environment. The study of optical properties in this work includes photoluminescence (PL) and absorbance spectra using a HITACHI F-7000 spectrophotometer.

## Results and discussion

**Fig. 2:**
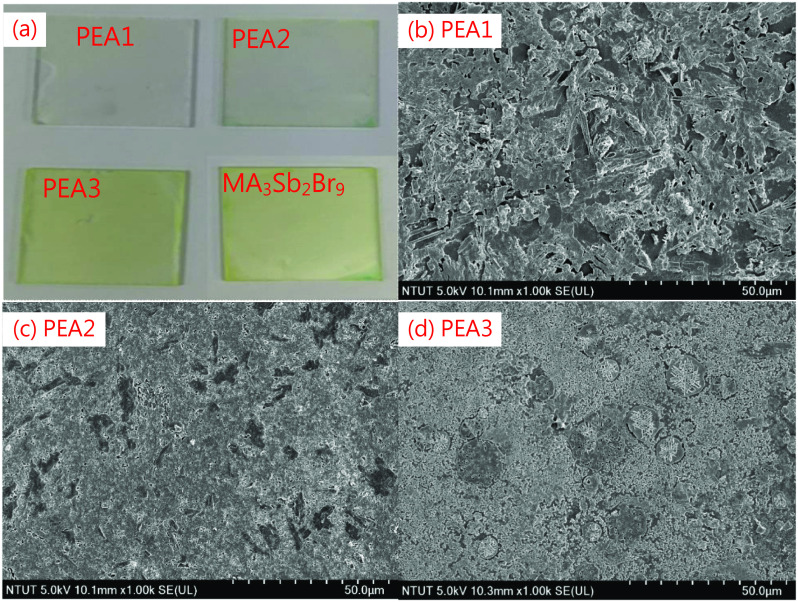
Pictures and top-view SEM images of (PEA)_2_MA_3_Sb_2_Br_9_ films with different amounts of PEABr

**Fig. 3:**
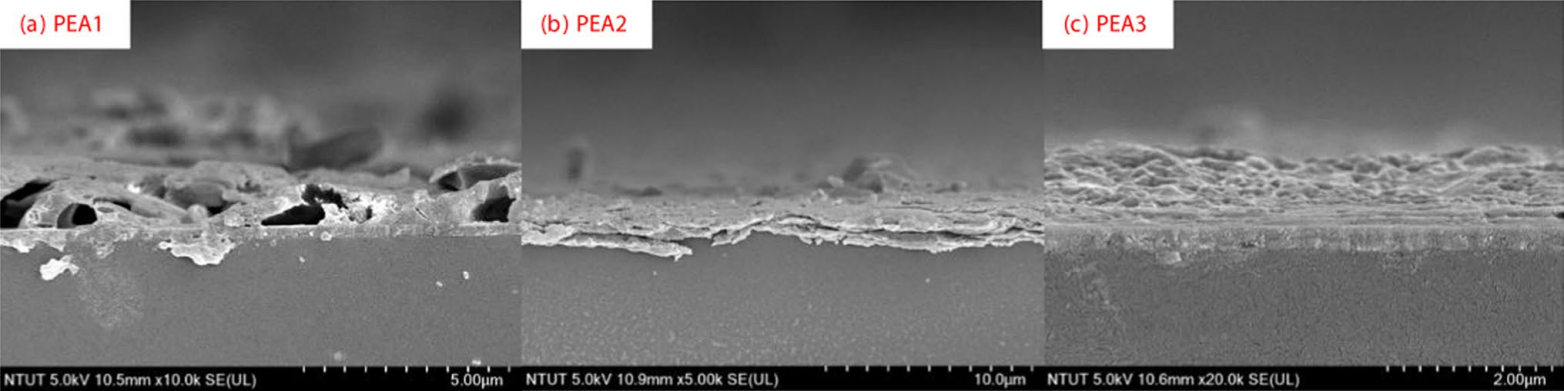
Cross-sectional SEM images of (PEA)_2_MA_3_Sb_2_Br_9_ films with different amounts of PEABr

Figure 2a shows the samples with various (PEA)_2_MA_3_Sb_2_Br_9_ films. More amount of MA_3_Sb_2_Br_9_ is present in the film, and more the sample look yellowish due to the MaBr band gap of 2.34 eV [29]. According to these SEM images, we noticed that with more PEABr, the surface morphology is rougher, as shown in Fig. 2b–d. For the cross-sectional SEM images, there are some holes on the surface of the PEA1 film, as show in Fig. 3a. However, as shown in Fig. 3b, c, for the samples of PEA2 and PEA3, layer-by-layer stacked 2D films without holes were observed.

**Fig. 4:**
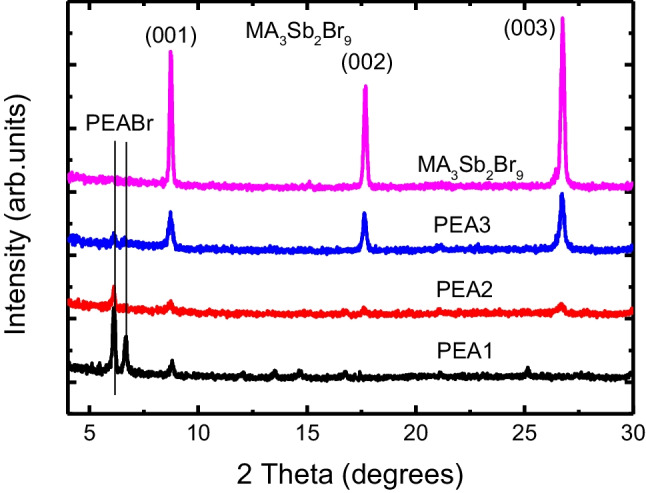
XRD patterns of the PEA1, PEA2, PEA3, and MA_3_Sb_2_Br_9_ films

To investigate the formation of (PEA)_2_MA_3_Sb_2_Br_9_ films, we performed grazing incidence X-ray diffraction (XRD) with wavelength of 1.54 Å. Figure 4 presents the XRD patterns for PEA1, PEA2, PEA3, and MA_3_Sb_2_Br_9_ films. In the XRD patterns, it can be indexed to trigonal symmetry with space group P3m1, and five peaks were observed. They are 6.1°, 6.7°, 8.7°, 17.7°, and 26.7°, respectively, corresponding to the (030) and (10-1) phases of PEABr and the (001), (002), and (003) phases of MA_3_Sb_2_Br_9_. The first two peaks of the XRD peaks are the 2D structure of PEABr [29–31].

**Fig. 5:**
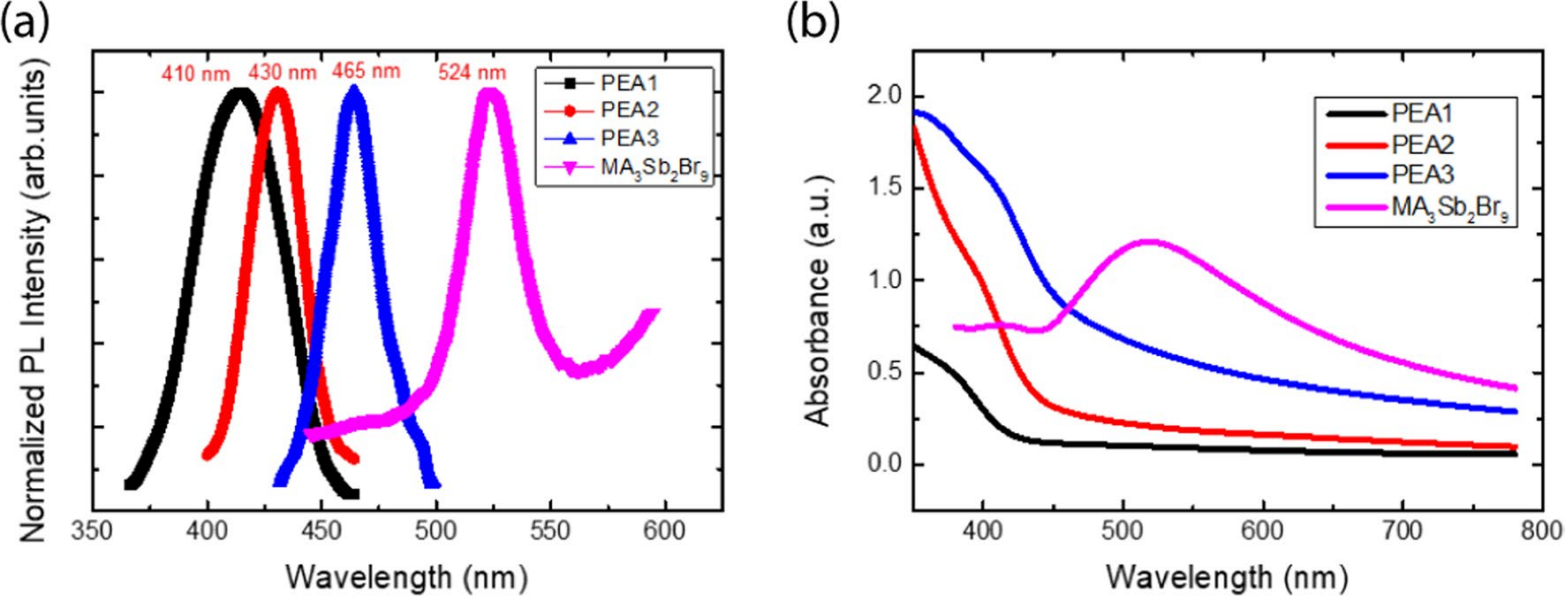
**a** Photoluminescence spectra (PL) and **b** absorption spectra of (PEA)_2_MA_3_Sb_2_Br_9_ films with different amounts of PEABr and MA_3_Sb_2_Br_3_ film

**Fig. 6:**
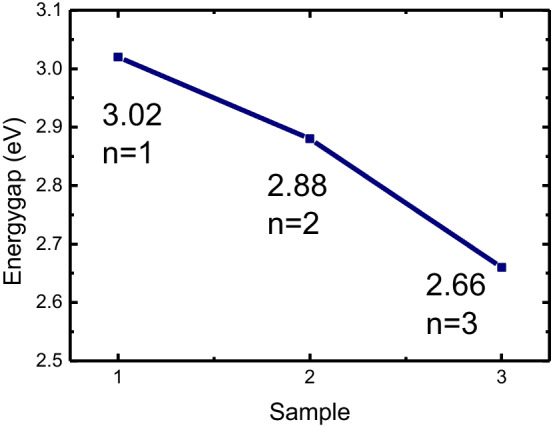
Bandgap of (PEA)_2_MA_3_Sb_2_Br_9_ films with different numbers of layers, n

The crystallinity and defect level in perovskite films can be observed by photoluminescence (PL). The PL emission spectra of the (PEA)_2_MA_3_Sb_2_Br_9_ films with different amounts of PEABr are shown in Fig. 5a. There is one PL emission peak for all samples. The peak positions for PEA1, PEA2, PEA3, and MA_3_Sb_2_Br_9_ are 420, 430, 465, and 524 nm, respectively. A sharp absorption edge was observed, as shown in Fig. 5b. The absorption positions of the PEA1, PEA2, PEA3, and MA_3_Sb_2_Br_9_ films are 420 nm, 440 nm, 470 nm, and 520 nm, respectively. They correspond to the energy gap. To calculate the band gap according to the PL and absorption spectra, the band gap is 3.02, 2.88, and 2.66 eV for samples of PEA1, PEA2, and PEA3, respectively, as shown in Fig. 6. The layer numbers are 1, 2, and 3 [30].

**Fig. 7:**
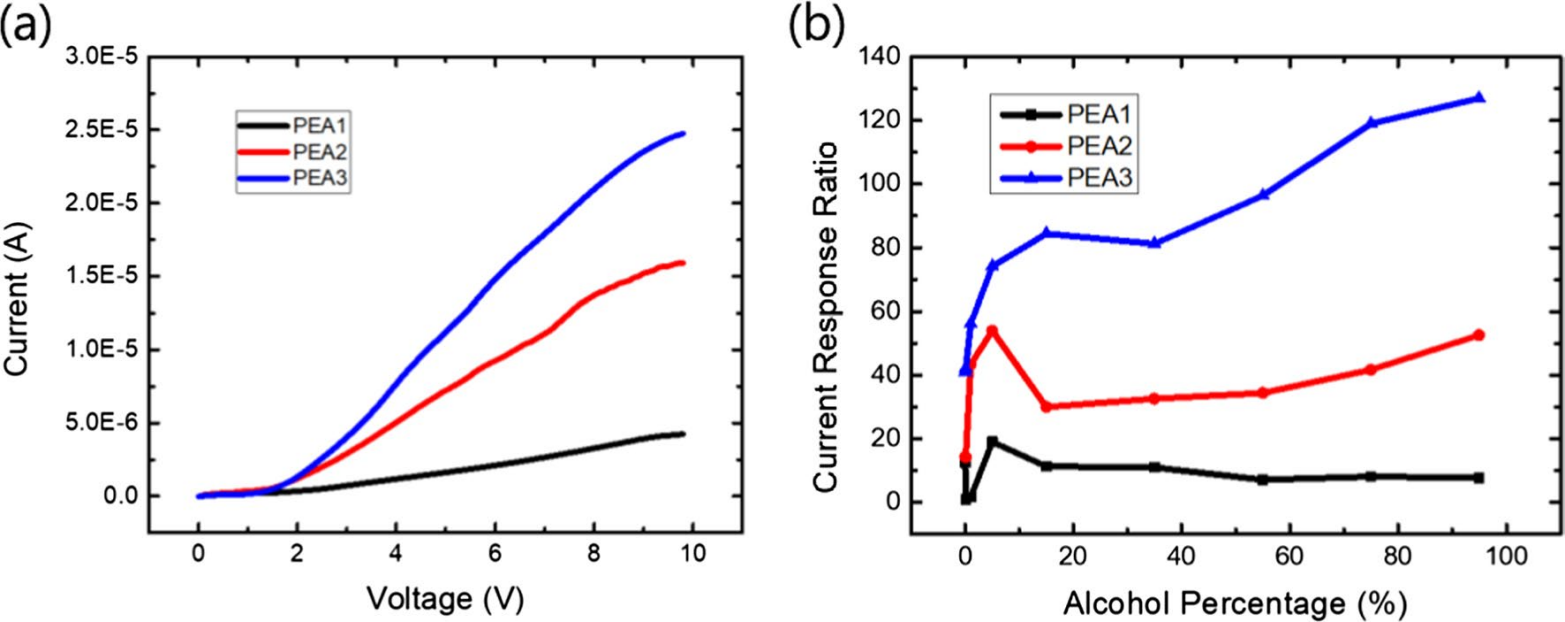
**a** Current–voltage characteristics and **b** current response ratio of (PEA)_2_MA_3_Sb_2_Br_9_ films with different alcohol concentrations

**Fig. 8:**
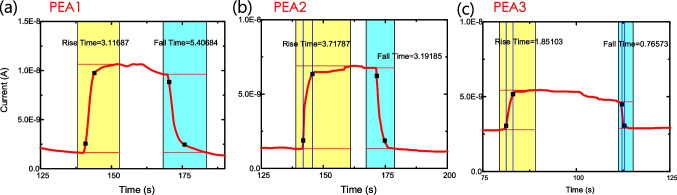
Current response of the (PEA)_2_MA_3_Sb_2_Br_9_ thin film alcohol detectors with different PEABr compositions: **a** PEA1: 0.4 mmol/ml, **b** PEA2: 0.2 mmol/ml, and **c** PEA3: 0.05 mmol/ml

Figure 7a shows the current–voltage (*I*–*V*) characteristics for PEA1, PEA2, and PEA3 samples in the ambient 5% alcohol concentration. We measured the *I*–*V* curves and estimated the current response ratio, as shown in Fig. 7b. All samples on the ambient alcohol with an alcohol concentration of 5% showed the highest current response ratio. For the PEA2 and PEA3 samples, as the alcohol concentration in the solution increases, the current response ratio still increases. This may be due to the dissolution of alcohol water and carbon dioxide induced by the catalyst effect of the quasi-2D (PEA)_2_(MA)_3_Sb_2_Br_9_ thin film. Figure 8 shows the current response of the (PEA)_2_MA_3_Sb_2_Br_9_ thin-film alcohol sensors with different PEABr compositions. The rise and fall times of the PEA3 alcohol detectors were 1.85 and 0.7 s, respectively, which were faster than the rise time (3.1 and 3.7 s) and fall times (5.4 and 3.2 s) of the PEA1 and PEA2 alcohol detectors, respectively. This indicates better conductivity and fewer surface states.

**Fig. 9:**
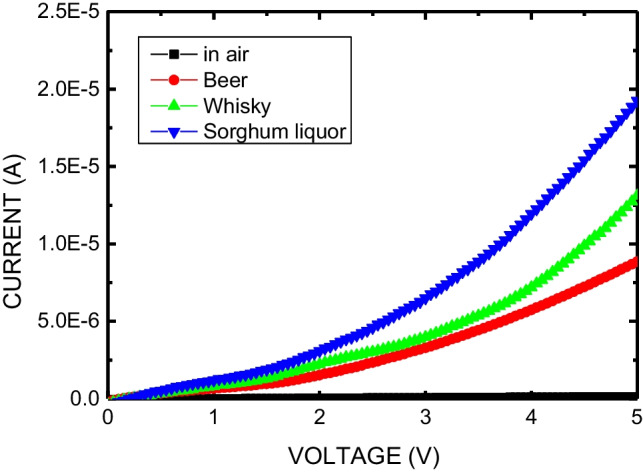
Current–voltage characteristics of PEA3 sample in various alcoholic beverage ambient

Finally, for real application, real sample characteristics using commercially available alcoholic beverage have presented in Fig. 9 to clarify the suitability of this work. The alcohol by volumes (ABV) of beer, whisky, and sorghum liquor are 5, 40, and 58%, respectively. As shown in Fig. 9, when the ABV increases, the resistance decreases. The current response ratio of beer, whisky, and sorghum liquor are 69, 103, and 152, respectively, for PEA3 sample. It may be contributed to the conductivity of the (PEA)_2_MA_3_Sb_2_Br_9_ film. Therefore, this work is suitable for the application of industry.

## Conclusions

In conclusion, we have studied the characteristics of (PEA)_2_MA_3_Sb_2_Br_9_ thin films and their application to alcohol detectors. The optimal current response ratios are 74 and 84 for 5 and 15% alcohol solutions, respectively. When the amount of PEABr decreases in the films, the conductivity of the sample in ambient alcohol with a high alcohol concentration solution increases. This may be due to the dissolution of alcohol into water and carbon dioxide induced by the catalyst effect of the quasi-2D (PEA)_2_(MA)_3_Sb_2_Br_9_ thin film. The situation in which the conductivity of the sample increased under ambient alcohol with increasing alcohol concentration in solution improved.

## Data Availability

All the data are fully available without restrictions.

## Abbreviations

QTD: Quasi-two-dimensional
(PEA)_2_(MA)_3_Sb_2_Br_9_: Palmitoylethanolamide methylammonium antinomy bromide
SbBr_3_: Antimony tribromide
MABr: Methylammonium bromide
DMF: Dimethylformamide
PEABr: Phenethylamine hydrobromide
FESEM: Field-emission scanning electron microscopy
ITO: Indium tin oxide
